# Chromatic Variants of Pityriasis Versicolor and Molecular Species Identification Using Polymerase Chain Reaction-Restriction Fragment Length Polymorphism (PCR-RFLP)

**DOI:** 10.3390/jof12030202

**Published:** 2026-03-11

**Authors:** Marina Romero-Navarrete, Francisca Hernández-Hernández, Roberto Arenas, Aureliano Castillo-Solana, Lizbeth Magnolia Martínez-Aguilar, Erika Córdova-Martínez, Brianda Stephanie Herrera-Ramírez, Settanan Plangsiri, Teerapong Rattananukrom

**Affiliations:** 1 Acapulco General Hospital IMSS Bienestar, Acapulco 39910, Guerrero, Mexico; marina.romero.n66@gmail.com (M.R.-N.); aurecastillo@hotmail.com (A.C.-S.); 2 Mycology Unit, Department of Microbiology and Parasitology, Faculty of Medicine, National Autonomous University of Mexico, Mexico City 09230, Mexico; micoher@hotmail.com (F.H.-H.); magnoliamarths@gmail.com (L.M.M.-A.); erikacmunica@yahoo.com.mx (E.C.-M.); briandahramirez@gmail.com (B.S.H.-R.); 3 Mycology Section, Hospital General “Dr. Manuel Gea Gonzalez”, Mexico City 14080, Mexico; rarenas98@hotmail.com; 4 Faculty of Medicine Ramathibodi Hospital, Mahidol University, Bangkok 10400, Thailand; settanan.pla@student.mahidol.edu; 5 Division of Dermatology, Department of Medicine, Faculty of Medicine Ramathibodi Hospital, Mahidol University, Bangkok 10400, Thailand

**Keywords:** *Malassezia* spp., molecular biology, PCR-RFLP, pityriasis versicolor, tinea versicolor

## Abstract

Background: Pityriasis versicolor (PV) is a common superficial mycosis caused by *Malassezia* species. To describe the clinical and epidemiological characteristics of PV in Acapulco, Mexico, and to identify the associated *Malassezia* species using polymerase chain reaction–restriction fragment length polymorphism (PCR-RFLP). Methods: A cross-sectional study was conducted in 2024 at Acapulco General Hospital and a private dermatology clinic. Patients with clinically suspected PV and no recent antifungal or immunosuppressive treatment were enrolled. Skin scales were examined microscopically and cultured on modified Dixon agar. Isolates were identified using conventional methods and PCR-RFLP with HhaI and BstCI enzymes. Results: Sixty-nine patients were included; 68.1% were male, and the most affected age group was 11–20 years (34.8%). The hypochromic variant predominated (63.8%). PCR-RFLP identified *M. globosa* (33.3%) and *M. furfur* (31.9%) as the most frequent species, followed by *M. restricta*, *M. sympodialis*, and *M. slooffiae*. Species identification was unsuccessful in 11.6% of isolates. No statistically significant associations were found between clinical variants, gender, or species distribution. Conclusions: *M. globosa* and *M. furfur* were the predominant species in this tropical Mexican cohort. PCR-RFLP is a practical option for species-level identification, highlighting the diversity of *Malassezia* in PV.

## 1. Introduction

Pityriasis versicolor (PV) is a common superficial mycosis of the stratum corneum caused by lipophilic yeasts of the genus *Malassezia*. Clinically, it presents as hypopigmented, hyperpigmented, erythematous, or mixed macules with fine scaling, predominantly affecting seborrheic areas such as the trunk, neck, and upper extremities. Facial involvement is more frequently observed in children. Although benign, PV is chronic and highly recurrent, with relapse rates reported to reach 60% within one year and up to 80% within two years [1,2].

PV is endemic in tropical and subtropical regions, where prevalence may reach 30–50% of the population, while it is considerably less frequent in temperate climates [3]. Predisposing factors include heat, humidity, increased sebaceous activity, hyperhidrosis, oily skin, use of topical oils or corticosteroids, and immunosuppression. The disease predominantly affects adolescents and young adults, likely reflecting increased sebum production after puberty, with a slight male predominance reported in some series.

Several *Malassezia* species have been implicated in PV, most commonly *M. globosa*, *M. furfur*, and *M. sympodialis*, although species distribution varies geographically. In addition, attempts to correlate clinical presentation, particularly pigmentary variation with specific *Malassezia* species have yielded inconsistent results, suggesting that host factors, environmental conditions, and methodological differences may influence observed patterns [4].

In Mexico, identification of *Malassezia* species has traditionally relied on phenotypic and biochemical methods, which may lack precision and reproducibility. Molecular approaches, such as polymerase chain reaction–restriction fragment length polymorphism (PCR–RFLP), provide a more accurate and reliable method for species-level identification [5]. Given the tropical climate of Acapulco and the limited molecular epidemiological data available for this region, this study aimed to describe the clinical and epidemiological characteristics of PV in a Mexican population and to identify the associated *Malassezia* species using PCR–RFLP with two restriction enzymes.

## 2. Material and Methods

A cross-sectional study was conducted in 2024 at the General Hospital of Acapulco and a private dermatology clinic, including patients of all ages and genders with clinically diagnosed PV and confirmed *Malassezia* isolates. Participants had not received immunosuppressants, corticosteroids, or topical/systemic antifungals within the past three months. The study was approved by the Institutional Review Board of the Secretary of Health in Guerrero, Mexico (Folio: 03140617).

Scales were obtained by scraping the affected skin and subjected to direct microscopic examination using either 20% potassium hydroxide or methylene blue, revealing characteristic short, thick filaments and yeast cells. Specimens were cultured on modified Dixon agar and incubated at 32 °C for up to three weeks. Cultures with colonies suggestive of *Malassezia* were examined microscopically using lactophenol blue to confirm yeast-like morphology. Gram-stained smears were performed to assess culture purity.

A total of 69 patients with *Malassezia* isolates were obtained for species identification. All isolates underwent both macroscopic and microscopic evaluation. Microscopically, *M. globosa* presented as clusters of round to oval yeast cells, whereas *M. furfur* showed budding yeast cells with occasional short filaments. Representative isolates were morphologically confirmed as *M.* globosa. and *M. furfur*, respectively. In addition, *M. restricta* showed small, round to oval yeast cells with occasional short, unbranched filaments, typically arranged singly or in pairs. *M. slooffiae* displayed ovoid to cylindrical yeast cells with polar budding; short pseudohyphae may be occasionally observed.

Cultures displaying two or more distinct colony morphologies were subcultured and analyzed separately. A catalase test was performed on all isolates; a catalase-negative result was considered highly suggestive of *M. restricta* [6]. In addition, cultures were inoculated on Sabouraud dextrose agar to detect *M. pachydermatis*, confirmed by positive growth. Pure isolates were subcultured by massive streaking on modified Dixon agar and incubated at 32 °C for 5–7 days to obtain sufficient biomass for DNA extraction.

All isolates and reference strains were initially identified using conventional phenotypic methods, including evaluation of colony morphology and lipid dependence on Dixon agar, catalase reaction, and microscopic examination of yeast cell shape and budding patterns. These preliminary phenotypic identifications were subsequently confirmed by PCR amplification of the 26S rDNA region followed by RFLP analysis.

### 2.1. DNA Extraction and PCR

The yeast mass was harvested directly from the agar. DNA extraction was then performed using either the phenol-chloroform method [7] or the GeneAll Exgene™ Plant SV Mini extraction kit (Cat. 117-101, Songpa-gu, Seoul, Republic of Korea). The extracted DNA was quantified and analyzed on a 0.8% agarose gel, stained with GelRed (Biotium, Hayward, CA, USA). A total of 50 ng of DNA was used for PCR amplification of the highly conserved 26S rDNA region using specific oligonucleotides: Forward (Fw): 5′-TAA CAA GGA TTC CCC TAG TA-3′ and Reverse (Rev): 5′-ATT ACG CCA GCA TCC TAA G-3′ [8]. The PCR reaction was performed in a 50 μL reaction volume containing 1× PCR buffer, 1.5 mM MgCl_2_, 0.2 mM dNTPs, 0.2 mM oligonucleotides, and 2.5 U recombinant Taq DNA polymerase (Thermo Scientific, Waltham, MA, USA). The PCR amplification conditions consisted of an initial denaturation at 94 °C for 5 min, followed by 30 cycles of denaturation at 94 °C for 45 s, annealing at 55 °C for 45 s, and extension at 72 °C for 1 min, with a final extension at 72 °C for 7 min. The amplified products were resolved on a 1.5% agarose gel, yielding a 600 bp fragment, which was subsequently purified using the DNA Clean & Concentrator-5™ kit (Zymo Research, Irvine, CA, USA) and quantified.

### 2.2. Restriction Fragment Length Polymorphism (RFLP)

The PCR products were digested with two restriction enzymes, HhaI and BstCI (New England BioLabs, Ipswich, MA, USA). The digestion reaction was performed in a final volume of 10 μL, consisting of 1× buffer, 200 ng of PCR product, and 10 U of restriction enzyme. The incubation conditions were set at 37 °C for *HhaI* and 65 °C for *BstCI*, with an optimized incubation time of 3 h for both enzymes. The digestion products were subsequently separated by electrophoresis on a 2.5% agarose gel at 75 V for 1.5 h. The resulting restriction patterns were analyzed and compared with previously published studies conducted under similar conditions, as reported in the literature [8].

**Figure 1:**
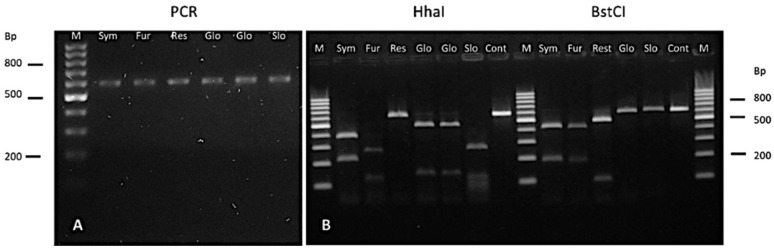
PCR and restriction fragment length polymorphism (RFLP) analysis of *Malassezia* reference strains were performed to compare them with the clinical isolates included in this study.; (**A**) PCR product; (**B**) Digestion products of reference strains treated with HhaI and BstCI enzymes. M: 100 bp molecular weight marker. Restriction patterns: Sym, *M. sympodialis* (400 and 220 bp); Fur, *M. furfur* (250 and 120 bp); Res, *M. restricta* (600 bp, undigested); Glo, *M. globosa* (450 and 130 bp); Slo, *M. slooffiae* (250, 110, and 80 bp); Cont: Undigested PCR product control.

PCR amplification yielded a 600 bp fragment in all isolates, similar to the reference strains. (Figure 1A). Species identification was subsequently confirmed by RFLP analysis, based on digestion patterns with HhaI and BstCI enzymes, and compared against reference strains (Figure 1B).

### 2.3. Statistical Analysis

Patient characteristics were summarized using the mean or median for continuous variables and frequencies with percentages for categorical variables. Comparisons across clinical outcomes were performed using the Chi-square test or Fisher’s exact test, as appropriate. All statistical analyses were conducted using STATA version 18.0. A *p*-value < 0.05 was considered statistically significant.

## 3. Results

### 3.1. Epidemiological Data

A total of 69 patients with clinically diagnosed PV were included, ranging in age from 2 months to 72 years. The majority of cases were observed in the 11–20-year age group (34.8%), followed by the 21–30-year (18.9%) and 0–10-year (17.4%) groups. The prevalence decreased markedly in older age groups, with only 7.2% of cases occurring in individuals aged 41–50 years and sporadic cases in those above 50 years of age. Males were significantly more affected than females, accounting for 68.1% of cases, while females comprised 31.9% (Table 1).

### 3.2. Clinical Type of Pityriasis Versicolor Associated with Malassezia Species

**Figure 2:**
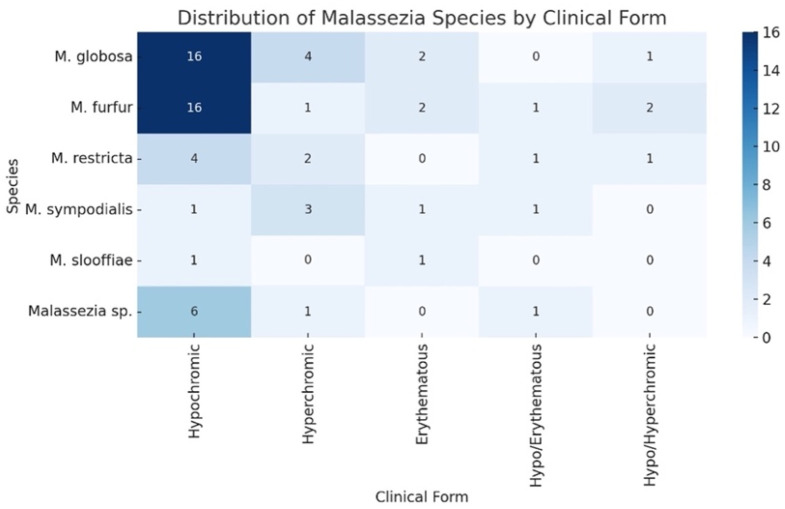
Distribution of *Malassezia* species by clinical form.

**Figure 3:**
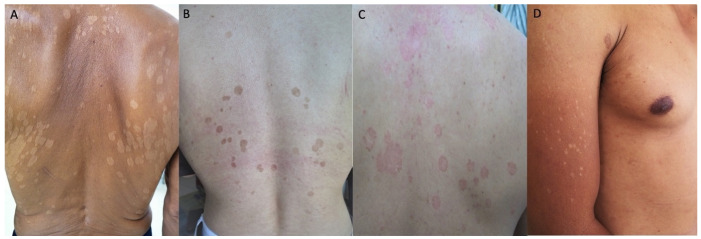
Clinical variants of pityriasis versicolor (**A**) Hypochromic; (**B**) Hyperchromic; (**C**) Erythematous; (**D**) Hypochromic–Hyperchromic.

The hypochromic type was the most prevalent clinical presentation, observed in 44 patients (63.8%), followed by hyperchromic (15.9%), erythematous (8.7%), hypochromic–erythematous (5.8%), and hypochromic–hyperchromic types (5.8%) (Figure 2 and Figure 3). When analyzed by species (column-based percentages), hypochromic lesions were most frequently observed in *M. furfur* (72.7%) and *M. globosa* (69.6%) isolates. Hyperchromic lesions were most commonly associated with *M. sympodialis* (50.0%), while *M. globosa* (17.4%) and *M. furfur* (4.6%) were less frequent. Erythematous lesions were identified in 50.0% of *M. slooffiae* isolates and 16.7% of *M. sympodialis* isolates. Mixed lesion types (hypochromic–erythematous and hypochromic–hyperchromic) were distributed across species without a clear predominance. Statistical analysis revealed no statistically significant association between clinical morphology and the isolated *Malassezia* species, with *p*-values greater than 0.05 using Fisher’s exact test. (Table 2).

### 3.3. Clinical Type of Pityriasis Versicolor Associated with Gender

Forty-seven (68.1%) were male and 22 (31.9%) were female. The hypochromic type was the most common presentation in both genders, observed in 32 males (68.1%) and 12 females (54.6%). Hyperchromic lesions were found in 6 males (12.8%) and 5 females (22.7%), while the erythematous type was noted in 3 patients in each group (6.4% in males and 13.6% in females). Hypochromic–erythematous lesions were observed exclusively in male patients (8.5%), whereas hypochromic–hyperchromic lesions occurred in 2 patients from each gender group. Statistical analysis showed no statistically significant association between gender and the clinical type of PV, with *p*-values greater than 0.05 using Fisher’s exact test (Table 3).

### 3.4. Association Between Malassezia Species and Gender

The distribution of *Malassezia* species according to gender is presented in Table 4. The most frequently identified species overall were *M. globosa* (33.3%) and *M. furfur* (31.9%). Among male patients, *M. furfur* was slightly more prevalent (36.2%) than *M. globosa* (29.8%). In contrast, *M. globosa* was more common in females (40.9%) than *M. furfur* (22.7%). Species-level identification was inconclusive in 11.6% of cases due to restriction patterns not matching any reference strain or previously reported species; these were classified as *Malassezia* spp. *M slooffiae* was detected only in female patients (9.1%), while no male patients yielded this species. However, statistical analysis showed no significant association between gender and the distribution of *Malassezia* species (*p* = 0.316).

Among the three infant cases (<1 year of age), *M. furfur* was identified in two patients (aged 2 and 8 months) presenting with hypochromic and hypochromic–erythematous forms, respectively, while *M. restricta* was isolated in a 3-month-old patient with a hypochromic–erythematous form.

## 4. Discussion

Since *Malassezia* was first identified as the causative agent of PV, its identification has primarily relied on phenotypic and biochemical methods. Until 1996, only three species were recognized: *M. furfur*, *M. sympodialis*, and *M. pachydermatis*. Due to the limitations of morphological and biochemical approaches, various molecular techniques have since been developed to improve species identification. In 1996, Guého et al. [6]. described four additional species—*M. globosa*, *M. slooffiae*, *M. restricta*, and *M. obtusa*—based on molecular methods. To date, 21 *Malassezia* species have been identified, with approximately 11 known to infect humans [2,9]. Molecular tools such as PCR-RFLP, nested PCR, multiplex PCR, real-time PCR, and sequencing have increasingly been adopted for accurate species-level identification [2,8,10,11,12,13,14,15,16,17,18,19,20,21,22].

Acapulco, located on the southern Pacific coast of Mexico, has a humid tropical climate with temperatures ranging from 21 °C to 34 °C, creating ideal conditions for the development of PV. This environmental context underscores the need for regional studies to better understand the disease and its etiologic agents.

In our study, the highest prevalence was found in the 11–20-year age group, differing from reports in Egypt, Argentina, and Iran, where the most affected age groups were between 20–30 years [8,12,23]. Similarly, a Brazilian clinical-epidemiologic study reported that PV was more prevalent around puberty, with the most affected age group in the 10–19 year range [24]. Another Brazilian outpatient series found the highest frequency in adolescents and young adults (10–20 years) [25]. In contrast, large retrospective laboratory-based data from southern Brazil show a median adult age in the early 30 s, reflecting a different healthcare setting and case mix [26]. Importantly, we documented three cases in infants under one year of age (2, 3, and 8 months), which, to our knowledge, is the first report in Mexico confirming *Malassezia* species identification in this age group using molecular methods. Prepubertal cases may be underrepresented in many clinic- or laboratory-based series dominated by adolescents and adults. Pediatric reviews indicate that PV can occur in young children; however, its detection largely depends on study design and recruitment setting. Males were significantly more affected than females, accounting for 68.1% of cases, while females comprised 31.9% (male-to-female ratio 2:1). These data indicate a predominance of PV among adolescents and young adults, particularly males, which may reflect the role of hormonal factors, increased sebaceous gland activity, and lifestyle or environmental exposures during this age period.

The post-pubertal increase in PV is largely attributed to changes in sebum quantity and composition that create a lipid-rich environment favorable for *Malassezia* growth. However, additional factors may contribute, particularly in younger individuals. The use of skin oils and emollients, high humidity, and frequent sweating may increase cutaneous lipids and promote *Malassezia* proliferation, especially in tropical settings [4,27]. Differences in skin microbiome and barrier maturity may also influence susceptibility. Thus, beyond pubertal sebum changes, exogenous lipid exposure and age-related skin physiology likely play a role.

PV is an endemic dermatosis in tropical regions, with diagnosis primarily based on clinical presentation. However, classification of clinical variants in the literature remains inconsistent. Lesions are generally categorized as hypopigmented, hyperpigmented, or mixed types [6,8,28], which aligns with the findings observed in our adult patients. In our study, five clinical variants were identified (Table 2). Among the three cases in children under one year of age, two presented with hypochromic-erythematous lesions, and one exhibited a hypochromic form.

Traditional identification methods for *Malassezia* rely on morphological and biochemical criteria. However, recent studies highlight the greater sensitivity and specificity of molecular techniques. For example, a study in Egypt reported 100% identification with PCR compared to 75% using phenotypic methods [8]. A systematic review of 22 studies across 18 countries showed that PCR-RFLP is commonly used for species identification [2,10,11,12,14,15,23,28,29,30,31,32,33]. Countries such as Argentina [23], Chile [31], Egypt [8], India [10], Iran [15], and Vietnam [2] have all implemented PCR-RFLP for this purpose.

Of the 21 recognized *Malassezia* species, 10 have been implicated in PV through molecular studies, including *M. globosa*, *M. restricta*, *M. sympodialis*, *M. dermatis*, *M. furfur*, *M. obtusa*, *M. slooffiae*, *M. yamatoensis*, *M. pachydermatis*, and *M. japonica* [33]. In previous study, six species were identified: *M. globosa*, *M. furfur*, *M. sympodialis*, *M. restricta*, *M. slooffiae*, and *M. dermatis*. In our study, the most frequently isolated species was *M. globosa*, consistent with findings from China [33], Greece [28], Iran [12,15,29], Israel [32], and Turkey [14]. Additionally, studies from China [9], Egypt [8], India [10], Iraq [11], Japan [30], and Vietnam [2] identified *M. furfur* as the predominant species, which is consistent with our finding *M. furfur* as a frequently isolated species. In contrast, *M. sympodialis* was the most common species reported in Argentina [23]. Geographic variations in *Malassezia* species distribution may reflect differences in climate, host genetics, skin microbiota, lifestyle, and diagnostic methodologies, all of which can influence species prevalence across regions.

In this study, *M. globosa* and *M. furfur* were identified as the predominant species associated with PV, particularly in hypochromic lesions, which represented the most common clinical form. A higher frequency was observed in male patients, especially those with hypochromic variants. *M. furfur*, *M. restricta* and *M. sympodialis* were more frequently isolated from males, these associations were not statistically significant. The data suggest a potential trend toward species- and sex-related predilections in PV, but further studies with larger sample sizes are needed to clarify these observations.

Several studies have investigated whether clinical types of PV, including pigmentation, correlate with *Malassezia* species, but findings remain inconsistent. Many report no clear association between species and lesion color, suggesting that host factors and study design may play a greater role than species alone. For instance, Park et al. found no significant species difference between hypopigmented and hyperpigmented lesions [34]. Gaitanis et al. observed associations between pigmented forms and female sex and suggested that strain-level differences may influence disease extent [28]. Similarly, Archana et al. and Krisanty et al. reported variable correlations between species and clinical features, highlighting geographic and methodological differences across studies [3,35].

Our PCR-RFLP approach using HhaI and BstCI showed limitations in identifying all isolates. Some restriction patterns did not match known profiles and were classified as *Malassezia* spp. Three isolates initially suspected to be *M. nana*, *M. dermatis*, and *M. sympodialis* were later confirmed by sequencing as *M. furfur*, likely reflecting intraspecies variability. This phenomenon has been previously reported by Boekhout et al. [36] using random amplified polymorphic DNA analysis, which demonstrated genetic diversity within *Malassezia* spp.

Numerous studies have employed PCR-RFLP for *Malassezia* detection, often targeting the 26S rDNA gene—consistent with our study [8,12,13,16]—or the ITS2 region and ITS3/ITS4 primers [10,16,21]. A key factor affecting PCR-RFLP performance is the selection and number of restriction enzymes. Most studies used one or two enzymes. For example, CfoI enabled the differentiation of five species: *M. globosa*, *M. furfur*, *M. restricta*, *M. sympodialis*, and *M. slooffiae* [12]. HhaI alone identified *M. globosa* and *M. restricta* [13]. In contrast, a combining ITS3/ITS4 with HinfI and AluI distinguished seven species [21].

The highest discriminatory power was achieved using 26S rDNA with HhaI and BstCI, which successfully identified 11 species: *M. furfur*, *M. sympodialis*, *M. globosa*, *M. restricta*, *M. slooffiae*, *M. obtusa*, *M. dermatis*, *M. japonica*, *M. yamatoensis*, *M. pachydermatis*, and *M. nana* [8]. This method, also used in our study, remains one of the most robust PCR-RFLP approaches. Overall, PCR-RFLP is a cost-effective, straightforward technique for differentiating multiple *Malassezia* spp.

*M. furfur* fungemia is an underrecognized invasive infection in immunocompromised patients, especially those with central venous catheters and TPN. In this case, a patient with T-ALL developed concomitant *Bacillus cereus* septicemia and *M. furfur* fungemia, which was missed by automated blood culture and molecular systems but identified by microscopy, lipid-supplemented culture, and MALDI-TOF MS. The report highlights the diagnostic limitations of routine systems and the need for improved rapid detection methods [37].

Culture-independent methods include conventional PCR, multiplex PCR, real-time PCR, and PCR sequencing. Conventional PCR is the most economical but has the lowest resolution [11,18,21]. For instance, ITS3/ITS4 primers detected only *M. furfur*, *M. pachydermatis*, and *M. globose* [11]. Other studies using multiple primers identified up to four species [18], while ITS2-targeted approaches failed to differentiate *M. restricta*, *M. slooffiae*, and *M. pachydermatis* [21]. Moreover, these methods often require multiple reactions, increasing time and cost compared to PCR-RFLP.

Multiplex PCR allows for the detection of multiple species using species-specific primers that produce distinct amplicon sizes [14,22]. One study used three primer sets across three reactions to differentiate 11 species [22]. While effective, this method is labor-intensive due to the need for multiple parallel reactions. While effective, this method is labor-intensive due to the need for multiple parallel reactions [17]. Its main advantage lies in detecting co-infections more efficiently than PCR-RFLP. PCR sequencing offers the highest accuracy and can differentiate strains within the same species by comparing sequences with reference databases [10,19,20]. However, the high cost and need for specialized equipment limit its routine use for diagnosing PV.

## 5. Conclusions

*Malassezia* spp. associated with PV were identified using PCR-RFLP, detecting five main species: *M. globosa*, *M. furfur*, *M. sympodialis*, *M. restricta*, and *M. slooffiae*. *M. globosa* and *M. furfur* were the leading causative agents of PV in this tropical Mexican cohort, particularly in hypochromic lesions. PCR-RFLP proved to be a reliable tool for species-level identification, highlighting the diversity of *Malassezia* spp. in clinical infections. Currently, no standardized molecular methodology exists for the identification of *Malassezia* species. Further studies are warranted to improve our understanding of the regional and national epidemiology of *Malassezia* infections and their clinical manifestations in PV.

## Figures and Tables

**Table 1:** Age distribution of patients with culture-confirmed pityriasis versicolor.

Age Range (Years)	Males	Females	Total (*n*, %)
0–10	9	3	12 (17.4)
11–20	17	7	24 (34.8)
21–30	6	7	13 (18.9)
31–40	4	4	8 (11.6)
41–50	4	1	5 (7.2)
51–60	3	0	3 (4.3)
61–70	3	0	3 (4.3)
>70	1	0	1 (1.5)
Total (*n*, %)	47 (68.1)	22 (31.9)	69 (100)

**Table 2:** Clinical type of pityriasis versicolor associated with *Malassezia* species.

Clinical Form	Total	*M. globosa*	*M. furfur*	*M. restricta*	*M. sympodialis*	*M. slooffiae*	*Malassezia* spp.	*p*-Value
	*n* = 69	*n* = 23	*n* = 22	*n* = 8	*n* = 6	*n* = 2	*n* = 8	
Hypochromic, *n* (%)	44 (63.8)	16 (69.6)	16 (72.7)	4 (50.0)	1 (16.7)	1 (50.0)	6 (75.0)	0.140 *
Hyperchromic, *n* (%)	11 (15.9)	4 (17.4)	1 (4.6)	2 (25.0)	3 (50.0)	0 (0.0)	1 (12.5)	0.122 *
Erythematous, *n* (%)	6 (8.7)	2 (8.7)	2 (9.1)	0 (0.0)	1 (16.7)	1 (50.0)	0 (0.0)	0.338 *
Hypochromic–Erythematous, *n* (%)	4 (5.8)	0 (0.0)	1 (4.6)	1 (12.5)	1 (16.7)	0 (0.0)	1 (12.5)	0.224 *
Hypochromic–Hyperchromic, *n* (%)	4 (5.8)	1 (4.4)	2 (9.1)	1 (12.5)	0 (0.0)	0 (0.0)	0 (0.0)	0.829 *

* Fisher’s exact test.

**Table 3:** Clinical type of pityriasis versicolor associated with gender.

Clinical Form	Total (*n* = 69)	Males (*n* = 47)	Females (*n* = 22)	*p*-Value
Hypochromic, *n* (%)	44 (63.8)	32 (68.1)	12 (54.6)	0.276
Hyperchromic, *n* (%)	11 (15.9)	6 (12.8)	5 (22.7)	0.310 *
Erythematous, *n* (%)	6 (8.7)	3 (6.4)	3 (13.6)	0.375 *
Hypochromic–Erythematous, *n* (%)	4 (5.8)	4 (8.5)	0 (0.0)	0.299 *
Hypochromic–Hyperchromic, *n* (%)	4 (5.8)	2 (4.3)	2 (9.1)	0.587 *

* Fisher’s exact test.

**Table 4:** *Malassezia* species associated with gender.

Species	Total (*n* = 69)	Males (*n* = 47)	Females (*n* = 22)	*p*-Value
*M. globosa*	23 (33.3)	14 (29.8)	9 (40.9)	0.316 *
*M. furfur*	22 (31.9)	17 (36.2)	5 (22.7)	
*M. restricta*	8 (11.6)	6 (12.8)	2 (9.1)	
*M. sympodialis*	6 (8.7)	5 (10.6)	1 (4.6)	
*M. slooffiae*	2 (2.9)	0 (0)	2 (9.1)	
*Malassezia* spp.	8 (11.6)	5 (10.6)	3 (13.6)	

* Fisher’s exact test.

## Data Availability

The original contributions presented in this study are included in the article. Further inquiries can be directed to the corresponding author.

## Abbreviations

26S rDNA	26S ribosomal DNA
580 bp	580 base pairs
DNA	Deoxyribonucleic Acid
HhaI and BstCI enzymes	Restriction endonucleases HhaI and BstCI
ITS	Internal Transcribed Spacer
*M.*	*Malassezia*
PCR	Polymerase Chain Reaction
PCR-RFLP	Polymerase Chain Reaction–Restriction Fragment Length Polymorphism
